# Micropropagation of *Citronella mucronata* D. Don, a Vulnerable Chilean Endemic Tree Species

**DOI:** 10.3390/plants11182425

**Published:** 2022-09-16

**Authors:** Francesca Guerra, Loreto Badilla, Ricardo Cautin, Mónica Castro

**Affiliations:** 1 Laboratorio de Propagación, Escuela de Agronomía, Facultad de Ciencias Agronómicas y de los Alimentos, Pontificia Universidad Católica de Valparaíso, San Francisco S/N La Palma, Quillota 2260000, Chile; 2 Altoverde Paisajismo, Quillota 2260000, Chile; 3 Escuela de Agronomía, Facultad de Ciencias Agronómicas y de los Alimentos, Pontificia Universidad Católica de Valparaíso, San Francisco S/N La Palma, Quillota 2260000, Chile

**Keywords:** naranjillo, in vitro culture, conservation, native plants, vulnerable species, LED lighting, growth regulators

## Abstract

*Citronella mucronata* (*C. mucronata*), a tree species endemic to Chile, has become threatened in its natural habitat and is currently listed as vulnerable. Tree population parameters have deteriorated due to indiscriminate logging and other anthropogenic activities, warranting research on mass propagation as a means of recovery. This study, unprecedented for this native species, has developed a successful method for its micropropagation. The objective was to establish a protocol for in vitro propagation of *C. mucronata* to produce large quantities of high-quality seedlings in an accelerated plant acquisition process. The best results were achieved by growing explants on Murashige and Skoog (MS) basal culture medium supplemented with 4.44 μM 6-benzylaminopurine (BAP) and 14.76 μM indole-3-butyric acid (IBA). Explant survival rate was 78 %, the average shoot length reached 3.2 cm, the number of lateral shoots was 3.9, and rooting rate was 60%. Furthermore, stimulation with red and blue light in a 1:2 ratio, supplemented with 14.76 μM IBA, improved the rooting rate to 93%. The survival rate of rooted explants reached 100% in the acclimatization stage when using peat and perlite substrate (1:1 v/v).

## 1. Introduction

*Citronella mucronata*, popularly known as “naranjillo”, belongs to the Icacinaceae family of the genus *Citronella* and is an endemic Chilean tree species with an extensive but uneven distribution range between the Coquimbo Region (30°40′ S) and the Araucanía Region (38°43′ S). It occurs both in the Andes mountains and the Coastal Range, at altitudes ranging from 25 to 1450 meters above sea level [1]. The conservation status of this species in Chile is currently classified as “vulnerable” [2], due to the indiscriminate felling of individuals located in exotic forest plantations; agricultural tasks; damage by pathogens, mainly by fungi (*Capnodium* sp.); skeletonizing insects; and threats from animals browsing their fruits, leaves, and shoots [1]. *C. mucronata* grows in sclerophyll forests, including evergreen shrublands and deciduous woodlands. It is a very shade-tolerant species, usually found as stand-alone individuals or forming small thickets on shady slopes or in humid areas [3,4]. It is a tree 10 m high, its leaves have a whole or toothed thorny margin, it blooms in spring, and its fruit is a purple drupe that is observed in summer.

This species is typically propagated by seeds, which are extracted from ripe fruit by removing the pulp. Seeds are usually sown in mid-fall and germinate in spring, with a typical germination rate of over 50%. The species is characterized by slow growth, reaching a height of only about 15 cm one year after sowing [5].

However, no experimental studies have been conducted to establish optimum temperature and humidity conditions for the preservation of viable propagules [6]. The vegetative propagation process in this species usually starts with 10 cm long cuttings harvested from new shoots in late summer. These are placed on a cool propagating bench of sand substrate with powdered rooting hormone under intermittent mist irrigation. Rooting takes about 3 months [5]. To the best of our knowledge, no information is available on the micropropagation of *C. mucronata*. Therefore, it is expected that the in vitro propagation method from nodal explants proposed in this study will contribute to the mass regeneration of this vulnerable species.

The high extinction rate of plant species worldwide and the need to care for our botanical heritage have made germplasm conservation an important undertaking. In vitro culture has emerged as a viable option for propagating plants whose conservation status is at risk [7]. The proposed technique would thus help to maintain the gene pool and preserve unique plant tissues to prevent the loss of natural heritage due to biotic or abiotic problems [8]. Micropropagation is used in a very wide range of species, including ornamental and forestry plants, as well as in the cryopreservation of valuable tree propagation material. A unique feature of micropropagation is its ability to rapidly produce uniform seedlings and the ability to generate disease-free plants throughout the year, regardless of season or climate [9]. However, maintaining the quality of in vitro plants requires conditions that promote the growth and development of seedlings, light being a fundamental factor through its quality, intensity, and the photoperiod required by each species [10,11]. While in vitro cultivation has long relied on fluorescent lamps with a wide wavelength range of 350–750 nm, this range is not necessarily optimum for plant development. Light-emitting diodes (LEDs) have recently started gaining popularity as an alternative because of their low thermal radiation, durability, low energy consumption, and the ability to tune the monochromatic wavelengths to the optimum light output needed for photosynthesis [12,13].

The objective of this study was to establish an in vitro propagation protocol that would allow for mass production of high-quality *C. mucronata* seedlings and accelerate the process of obtaining plants.

## 2. Results

### 2.1. In Vitro Establishment

Explant survival rate on MS basal culture medium was 79% (*p ≤* 0.05) (Table 1). Treatment T3, conducted with half the concentration of MS basal medium macroelements, had more oxidized explants (44%) than treatments T1 and T2 (5% and 17%, respectively) (*p ≤* 0.05). The explants that were considered oxidized had a dark brown coloration, which later turned black, finally resulting in the death of the plant material. Bacterial contamination was the same among the three treatments (*p ≤* 0.05). However, fungal contamination was significantly higher in treatment T2 (17%) (*p ≤* 0.05) compared to treatments T1 and T3 (6% and 11%, respectively).

### 2.2. In Vitro Shoot Multiplication

**Figure 1:**
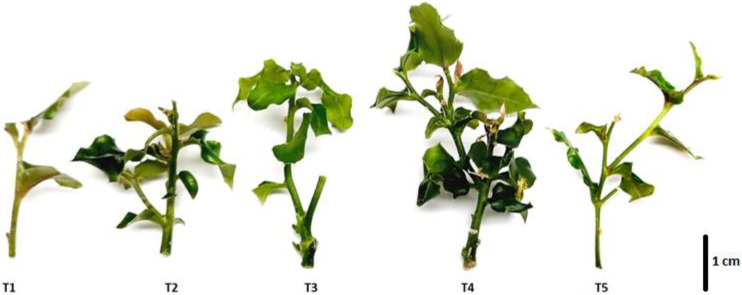
Representative shoots of *C. mucronata* at the in vitro multiplication stage under different experimental treatments. T1: Control; T2: 1.78 μM BAP; T3: 3.55 μM BAP; T4: 4.44 μM BAP; T5: 11.10 μM BAP. Scale bar 1 cm.

Based on the mean comparison test, the effect of different concentrations of 6-benzylaminopurine (BAP) (Table 2) on shoot growth was established. In uniform 1.5 cm shoots grown in vitro for three weeks and then transferred onto growth medium (MS basal medium with 0.05 μM IBA) supplemented with BAP, shoot length and height increased in all the treatments (Figure 1). The highest growth rate was achieved at 4.44 μM of BAP, with mean shoot height reaching 4.0 cm after 30 days (*p ≤* 0.05). Notably, shoot height increased by more than 1.5 cm in all the treatments. The use of BAP also led to an increase in lateral shoot length, with the best results obtained at the 4.44 μM concentration, where the average lateral shoot length reached 3.2 cm (*p ≤* 0.05).

Concentrations of 3.55 μM and 4.44 μM of BAP resulted in the highest average number of lateral shoots, 3.6 and 3.9, respectively (*p ≤* 0.05). The results of this experiment indicate that MS basal medium supplemented with BAP was successful in *C. mucronata* shoot multiplication.

### 2.3. In Vitro Rooting

**Figure 2:**
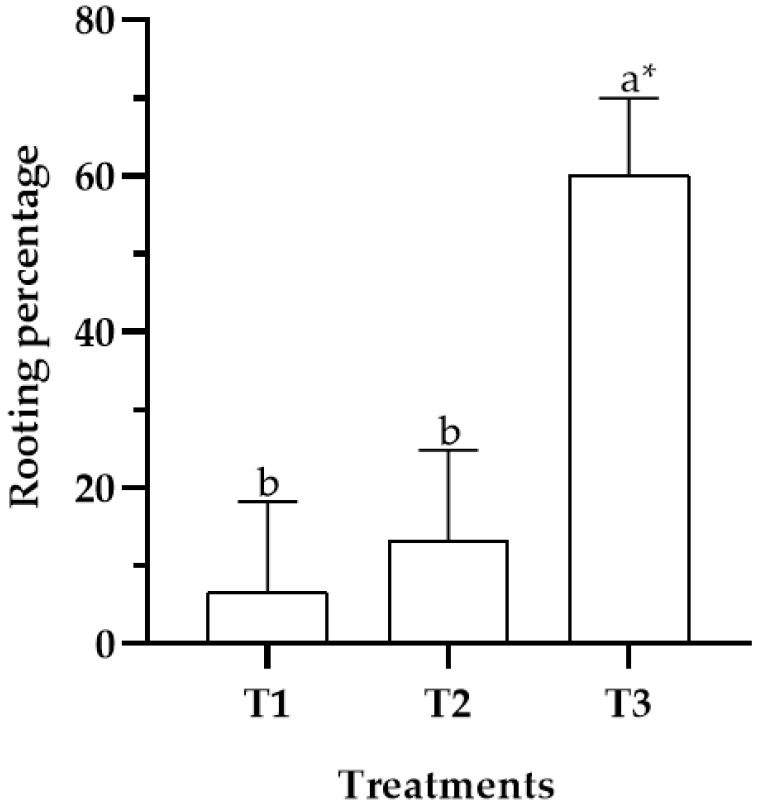
Rooting rate (%) of *C. mucronata* vitroplants under different concentrations of IBA in basal medium MS. T1: 0 μM IBA; T2: 4.92 μM IBA; T3: 14.76 μM IBA. * Letters indicate significant differences according to Tukey’s test (*p* ≤ 0.05). Bars represent the standard deviation.

The mean comparison test showed that the greatest effect on rooting was obtained on MS basal medium supplemented with IBA at a concentration of 14.76 μM (*p ≤* 0.05), which resulted in a rooting rate of 60% (Figure 2). In contrast, treatments T1 and T2 showed inferior rooting rates of 7% and 13%, respectively (*p ≤* 0.05).

### 2.4. Effect of Light Type on Rooting

According to measurements of yield photon flux density (YPFD, μmol m^−2^ s^−1^) of different light sources (Table 3), L2 yields 7 and 2 times more blue photons than L1 and L3, respectively. The yield of photosynthetically active radiation (PAR) is lower in L1 than in L2 or L3. The ratio of red to blue photons in L1 and L3 is 1:1, while in L2 it is 1:2. Providing the integral daily light (DLI) of the light sources is useful since it standardizes the light on the plants for a 24-hour period. In this case, L2 is the light source with the most photons per area, that is, it has a higher DLI than the other light sources.

**Figure 3:**
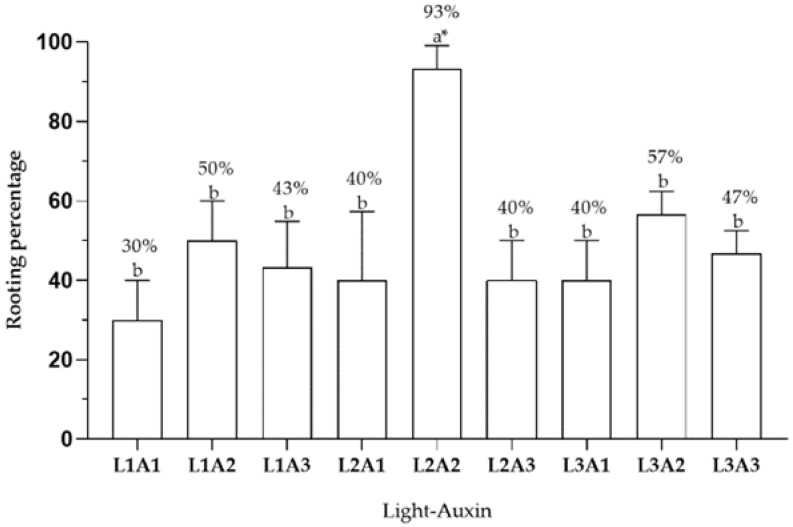
Effect of light type and IBA concentrations on rooting in *C. mucronata* vitroplants. L1A1: White light and 0 μM IBA; L1A2: White light and 14.76 μM IBA; L1A3: White light and 29.52 μM IBA; L2A1: Red:blue light 1:2 and 0 μM IBA; L2A2: Red:blue light 1:2 and 14.76 μM IBA; L2A3: Red:blue light 1:2 and 29.52 μM IBA; L3A1: Red:blue light 1:1 and 0 μM IBA; L3A2: Red:blue light 1:1 and 14.76 μM IBA; L3A3: Red:blue light 1:1 and 29.52 μM IBA. * Letters indicate significant differences according to Tukey’s test (*p* ≤ 0.05). Bars represent the standard deviation.

**Figure 4:**
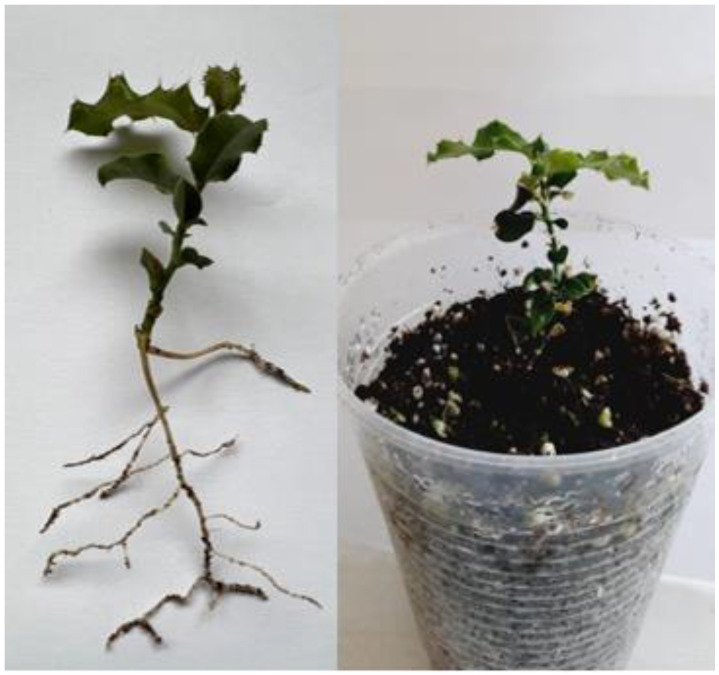
Rooted and acclimated *C. mucronata* plant.

The results summarized in Figure 3 show that the rooting rate (%) was significantly dependent on the type of light and auxin use (*p* ≤ 0.05). Thus, the combination L2A2 (red:blue 1:2; 14.76 μM of IBA) resulted in a 93% rooting rate of *C. mucronata*, while treatments without exogenous auxin (L1A1, L2A1, and L3A1) resulted in rooting rates of 30–40%. The use of auxin with all three light types (L1A2, L1A3, L2A2, L2A3, L3A2, and L3A3) improved rooting rates by more than 40% and by more than 50% at concentrations of 14.76 μM IBA (L1A2, L2A2, and L3A2). These results demonstrated that successful rooting in *C. mucronata* shoots depends on the presence and concentration of exogenous auxin, which favors all three types of light. All rooted plants subsequently transferred to the cold greenhouse for acclimation in a substrate with a 1:1 (v/v) peat to perlite ratio exhibited 100% survival rate after 30 days (Figure 4).

## 3. Discussion

The conservation status of *C. mucronata* in Chile is currently classified as “vulnerable” [2]. The aim of this current study was to develop the first protocol for mass production of this species by micropropagation This practice has become increasingly popular for conserving the gene pools of endangered species because it can rapidly produce high-quality seedlings [8]. Considering that this is the first scientific article that describes a protocol for the micropropagation of this tree species, only BAP was used as cytokinin and IBA as auxin. In the proposed protocol, the establishment stage of plant tissue was performed on MS, WPM, and MSm basal media, achieving 78% live explants on MS medium. The number of oxidized explants was lowest on MS and WPM media (17% and 5%, respectively). It should be noted that this oxidation, which began in the basal area of the explant, ended in the death of the plant material. Thus, within the treatments evaluated in this trial, when using the MS medium, the best response to induce the growth and development of explants from *C. mucronata* nodal segments was obtained.

The medium plays an important role in in vitro propagation by providing macro and micronutrients to the plant and by controlling the growth and development rate of the plant tissues by means of growth regulators [16]. One of the challenges of in vitro methods, especially for woody plants, is the risk of fungal or bacterial surface contamination when the complex procedure of explant disinfection is not performed correctly. Thus, the success of in vitro propagation depends on parameters such as the physiological state of the mother plant, the type of explant, the type and concentration of sterilizing agents, and sterilization exposure time. In addition, some of the sterilizing agents can be toxic to plant tissue, jeopardizing the success of the process [17]. Our protocol for disinfection of “naranjillo” explants guaranteed plant survival rates of over 80%, with the MS culture medium used in the establishment stage. These results are comparable to a study performed on *Crocus vernus* species, where 100% survival was obtained after disinfecting explants with 2% NaClO and 0.01% HgCl_2_ under constant agitation for 10 minutes [18].

However, even with optimum basal media, many genetic, biological, and ecological factors can interfere with the growth performance of species in the process of in vitro propagation [19]. In this study, “naranjillo” explants performed best on MS basal medium supplemented with the growth regulator BAP. This is the most widely used growth regulator in the industry to induce shoot buds in nodal segments during in vitro propagation [20]. Recent studies on *Azadirachta indica*, a tree with high medicinal value, reported maximum shoot proliferation and leaf numbers on MS culture medium supplemented with 2.22 μM BAP [21]. These results are comparable to our study, where the use of cytokinin (4.44 μM BAP) increased the average height of the explants from 1.5 cm to 4.0 cm. In *Daphne mezereum* L., the use of MS culture medium resulted in the maximum number of shoots and shoot length in combination with exogenous cytokinin at a concentration of 4.44 μM benzyladenine [22]. Thus, the composition of the culture medium is not the only factor that determines the success or failure of in vitro propagation. Cytokinins also play an important role as they control morphogenetic processes, such as the appearance of meristems. This is precisely why high concentrations of cytokinins and low concentrations of auxins are required for successful shoot proliferation [16]. 

Another factor to consider is the application of exogenous auxins to solve the problem of root induction in species with rooting difficulties [23]. This is a very serious problem in the micropropagation of many woody plants due to their limited physiological ability to form adventitious roots. Factors such as the use of adult material, secretion of inhibitors, and accumulation of ethylene during rooting can all negatively affect root formation [24]. Root induction and proliferation are affected not only by the type of auxin but also by the concentration and the duration of the auxin treatment, as well as by the culture medium [25]. In our experiments, rooting increased by 40–50% when IBA was used at a concentration of 14.76 μM in the culture medium. In contrast, when no exogenous auxin was applied, the rooting rate was only 7%. Other studies in woody species have also reported higher rooting rates with auxin: in *Quercus aliena*, the highest rooting rate (41%) was achieved with a combination of 4.92 μM IBA and 5 g L^−1^ activated carbon [26]. In the woody species *Acacia confusa*, 72% rooting rate was achieved by supplementing the MS culture medium with 4.92 μM IBA [27]. Rooting rates of 67 to 97% were obtained in species of the genus *Castanea* spp. with concentrations of 2.46 μM IBA [28]. The same effect was recorded in *Rhododendron wattii*, where root induction increased with 2.45 μM IBA [29]. In *Vaccinium corymbosum*, root induction was highest at concentrations between 2.46 μM and 4.92 μM IBA, attaining 84 and 100%, respectively [30].

It is worth noting that this is the first published study on *Citronella mucronata* grown under light-emitting diodes (LEDs). The use of LEDs for in vitro cultivation is an alternative to conventional white-light systems and benefits plants by providing optimum lighting conditions and light quality for each species [31,32]. The blue- and red-light spectra ranging from 460 nm to 660 nm are easily absorbed by chlorophyll and thus increase the efficiency of photosynthesis [31,32,33]. The boost received by plants from optimum lighting is especially useful in micropropagation, which is highly sensitive to a variety of factors, including the choice of species, the stage of plant tissue development, and the composition of the culture medium [33]. In this study, LED lighting was used primarily at the rooting stage; it was estimated that the use of LED lighting and the application of auxin growth regulator (14.76 μM IBA, red:blue LED ratio 1:2) resulted in 93% rooting in “naranjillo”.

On the other hand, white artificial lights have a complex absorption spectrum, due to the composition of blue, green, yellow, orange, red and far-red photons, which leads to the DLI and the μmol m^−2^ s^−1^ values received by the vitroplant being lower than the other sources of light (Table 3). In addition, it was observed that the use of IBA in a concentration of 14.76 μM with the different sources of lights achieved rooting percentages of 93% (L2), 57% (L3), and 50% (L1).

The effectiveness of LEDs at the rooting stage has also been confirmed in other studies. For example, a combination of white, red, and blue spectra resulted in a 4.5-fold increase in rooting in *Vaccinium* spp. compared to the use of white spectrum alone [34]. However, in the *Vanilla planifolia* species, the highest root counts were obtained with a combination of fluorescent (400–700 nm) and red LED lighting (660 nm), while using LED lighting alone increased shoot height, number of roots per plantlet, and number of leaves, but did not affect stomatal density [35]. Lighting systems with a 1:1 ratio of blue to red or biased toward the red spectrum inhibit root elongation, as this response is specifically mediated by red light receptors (phytochromes A and B). Conversely, white light promotes plant rooting due to the low proportion of radiation in the red region [36,37]. However, in *Protea cynaroides* species, a higher proportion of blue light enhanced the biosynthesis of phenolic compounds that inhibit in vitro rooting, while red light reduced the accumulation of 3,4-dihydroxybenzoic acid, gallic acid, and ferulic acid and thus promoted root formation [38,39]. The benefits of blue:red LED lighting systems are produced by the interaction between cryptochrome (blue light photoreceptor) and phytochrome (red light photoreceptor) pigments that mediate rhizogenesis through phytohormonal pathways [40]. The extensive advantages of LED lighting systems already documented will continue to drive research into their use for in vitro plant tissue culture, secondary metabolite production, photosynthesis, cell wall biosynthesis, and in vitro propagation of woody species [41,42,43].

For future studies, the use of other growth regulators and their combinations should be considered in order to improve the proliferation rates that allow optimizing the in vitro propagation protocol of this species. In addition, tests with material collected from wild populations should be included. On the other hand, the effect of light on the rooting of *C. mucronata* shoots propagated in vitro and the primary and secondary metabolism involved in the process should be investigated.

## 4. Materials and Methods

*Plant Material*

The *C. mucronata* explants were obtained from 3-year-old plants from the collection of this species consisting of 10 plants at the Propagation Laboratory of the Pontificia Universidad Católica de Valparaiso, Quillota, Chile. The seeds that gave rise to these plants were collected in the Cordillera del Melón, located northwest of Valparaiso, between 32°30′ and 32°46′ south latitude and 71° and 71°13′ west longitude. Plants were grown in a cold greenhouse in Quillota (32°54′ S, 71°16′ W) and were in full vegetative growth stage at the time of explant harvesting. Quillota is characterized by a warm temperate climate with semiarid humidity [44]. Prior to in vitro establishment, the plants were treated with antifungal and bactericidal solutions (Benlate^®^ 1.8 g L^−1^ and Phyton 3 cc L^−1^) every five days for 15 days.

*Disinfection of Plant Material*

Twenty-five young branches of *C. mucronata* were randomly selected, stripped of their leaves, washed under running water for 10 min, and rinsed with a sponge and Tween 20 (0.1%). Nodal segments 1.5 cm long with axillary buds from near the shoot tip were used as explants.

The explants were placed in an Erlenmeyer flask, covered with a mesh, and kept under continuous running water for 15 minutes. Next, the explants were disinfected in a 1% sodium hypochlorite (NaClO) solution with the antioxidants ascorbic acid 2838.97 μM and citric acid 2602.49 μM and a drop of Tween 20 under continuous agitation for 10 minutes. The material was then transferred to a laminar flow hood and rinsed four times with sterile distilled water. After disinfection, in vitro establishment of the plant material was initiated.

### 4.1. In Vitro Establishment

Nodal segments of *C. mucronata* were cultured in vitro and the effect of culture medium composition on viability (%) was evaluated as a percentage of live explants relative to the total number of explants per treatment. Other important aspects of explant establishment, such as contamination and oxidation, were also evaluated.

Thus, the experiment consisted of the following three treatments: (T1) MS basal medium [14]; (T2) WPM basal medium [15]; and (T3) modified MS medium (MSm) (half-strength MS medium macroelements, full-strength MS medium microelements and vitamins). In all the treatments, growth media were supplemented with 0.38 μM thiamine and 30 g L^−1^ sucrose. The pH of the media was adjusted to 5.7 ± 0.1 and 7 g L^−1^ agar was added. The media were sterilized in an autoclave for 15 min at 121 ºC. Once plantlets were established, they were transferred to a controlled-environment growth chamber with the temperature at 25 °C ± 1 °C, a photoperiod of 16:8 h (day:night), and fluorescent lamps (Philips TL-D 36W/54) providing cool white light (400–700 nm) with a YPFD of 9.96 μmol m^−2^ s^−1^. After 10 days of culture, evaluations were performed. Twenty-one random explants were used per treatment with three replicates each. Percentage values were transformed by natural logarithm. A one-way analysis of variance (ANOVA) and an analysis of variance components were performed. To establish differences between treatments, Tukey’s test (*p* ≤ 0.05) was performed using the Minitab statistical software (Minitab Inc., State College, PA, USA).

### 4.2. In Vitro Shoot Multiplication

The purpose of this experiment was to determine the effect of five concentrations of 6-benzylaminopurine (BAP) on shoot multiplication (0, 1.78, 3.55, 4.44, and 11.10 μM BAP). Shoots of uniform length (1.5 cm) from the establishment stage were subcultured on MS basal medium supplemented with 30 g L*^−^*^1^ sucrose and 0.49 μM indole-3-butyric acid (IBA). The pH of the media was adjusted to 5.7 ± 0.1 and 7 g L^−1^ agar was added. The explants were incubated in a growth chamber under the same conditions as above. After 30 days of in vitro culture, the height (cm) of the explants and the number of lateral shoots that emerged were evaluated. The experiment was conducted in triplicate of 10 explants each. A one-way analysis of variance (ANOVA) and an analysis of variance components were performed. To establish differences between treatments, Tukey’s test (*p* ≤ 0.05) was performed using the Minitab statistical software (Minitab Inc., State College, PA, USA).

### 4.3. In Vitro Rooting

The trial was carried out using uniform shoots (3 cm long) from the multiplication stage. Three concentrations of MS basal medium supplemented with IBA (0, 4.92, and 14.76 μM) were evaluated. Plant material was kept in a controlled environment growth chamber with a temperature of 25 °C ± 1 °C, photoperiod of 16:8 h (day:night), and fluorescent lamps (Philips TL-D 36W/54) providing cool white light (400–700 nm) with a YPFD of 9.96 μmol m^−2^ s^−1^. Ten explants were used per treatment with three replicates each. The evaluation criterion was rooting percentage, considering the number of rooted explants (0.5 cm root length) relative to the total number of explants in each treatment. A one-way analysis of variance (ANOVA) and an analysis of variance components were performed. To establish differences between treatments, Tukey’s test (*p* ≤ 0.05) was performed using the Minitab statistical software (Minitab Inc., State College, PA, USA).

### 4.4. Effect of Light Type on Rooting

This experiment used uniform 3 cm long explants from the in vitro proliferation stage to investigate the effects of three light types: L1 (white light (Philips TL-D 36W/54 cool fluorescent lamps)), L2 (red and blue light, 1:2 ratio (PARALED_FRRB 55W PARALED_FRRB system, Ciencia Pura SpA. Santiago, Chile)), and L3 (red and blue light, 1:1 ratio (PARALED PARALED_FRRB 55W system, Ciencia Pura SpA. Santiago, Chile)) and three auxin concentrations: A1 (0 μM IBA), A2 (14.76 μM IBA), and A3 (29.52 μM IBA) on *C. mucronata* in vitro rooting medium. The MS medium was used, and the plants were kept in a growth chamber at a temperature of 25 °C ± 1 °C with a 16:8 h (day:night) photoperiod.

In this experiment, types of light with different ratios of blue (420–460 nm) to red (660 nm) were used. To avoid light contamination, each treatment was separated by a white screen. The evaluation parameter was rooting rate (%) based on the number of rooted explants (with roots of at least 0.5 cm long) relative to the total number of explants per treatment. All rooted plants were subsequently transplanted into 200 cc containers with peat/perlite mixture (1:1 v/v) and transferred to a cold greenhouse for acclimation. Yield photon flux densities (YPFD, μmol m^−2^ s^−1^) and red:blue ratios were measured using a spectrophotometer (Lighting Passport Standard Pro model, Allied Scientific ProTM, Gatineau, QC, Canada). The design of the experiment was completely randomized with a 3x3 factorial arrangement. Ten explants per treatment were used with three replicates each. A two-way analysis of variance (ANOVA) and an analysis of variance components were performed. To establish differences between treatments, Tukey’s test (*p ≤* 0.05) was performed using the Minitab statistical software (Minitab Inc., State College, Pennsylvania, USA).

## 5. Conclusions

This is the first reported protocol of in vitro propagation of *C. mucronata* by successfully establishing explants from nodal segments on MS basal medium. Supplementation of the basal medium with BAP boosted plantlet height and the number of shoots per explant. Adding IBA to the culture medium improved the rooting rate of *C. mucronata* by 40–50%. Rooting induction and propagation benefited greatly from the use of LED lights with a red:blue ratio of 1:2 and supplementation with 14.76 μM IBA. Greenhouse acclimation of plantlets rooted on peat and perlite substrate was 100% successful. Thus, the micropropagation protocol developed in this study provides a tangible opportunity for mass multiplication of this vulnerable species. The results of this study may be relevant for the development of conservation strategies for *C. mucronata* and other woody species.

## Figures and Tables

**Table 1:** Establishment of *C. mucronata* in vitro on different culture media.

Treatment	Survival (%)	Oxidation (%)	Fungal Contamination (%)	Bacterial Contamination (%)
T1: MS	79 ± 2.3 a*	5 ± 1.3 b	6 ± 1.7 b	11 ± 1.1 a
T2: WPM	50 ± 2.8 b	17 ± 1.7 b	17 ± 2.7 a	17 ± 2.2 a
T3: MSm	35 ± 2.5 b	44 ± 1.7 a	11 ± 2.4 b	11 ± 2.0 a

* Letters indicate significant differences according to Tukey’s test (*p ≤* 0.05). Mean ± standard deviation. T1: Murashige and Skoog medium (MS) [14]; T2: Woody plant medium (WPM) [15]; T3: MSm (modified MS medium—half-strength MS medium macroelements, full-strength MS medium microelements and vitamins).

**Table 2:** Effect of different concentrations of BAP on in vitro *C. mucronata* shoot multiplication using 1.5 cm long explants.

Treatment	Concentration BAP (μM)	Shoot Length (cm)	Lateral Shoot Length (cm)	Number of Lateral Shoots
T1	0	3.0 ± 0.6 bc*	2.0 ± 0.5 cd	2.0 ± 1.2 b
T2	1.78	3.3 ± 0.5 bc	2.4 ± 0.4 bc	2.2 ± 1.3 b
T3	3.55	3.4 ± 0.2 b	2.6 ± 0.2 b	3.6 ± 1.4 a
T4	4.44	4.0 ± 0.3 a	3.2 ± 0.4 a	3.9 ± 1.6 a
T5	11.10	3.0 ± 0.6 c	1.9 ± 0.6 d	2.1 ± 1.2 b

* Letters indicate significant differences according to Tukey’s test (*p ≤* 0.05). Mean ± standard deviation.

**Table 3:** Red and blue yield photon flux densities (YPFD, μmol m ^−2^ s^−1^) and photon ratios of different types of lights.

Light Type	Red	Blue	Red:Blue Ratio	DLI (mol m^−2^)
(YPFD, μmol m^−2^s^−1^)				
L1	2	2	1:1	0.8
L2	7	14	1:2	3.8
L3	6	6	1:1	2.1

s: second, m: meter.

## Data Availability

Not applicable.
